# 

**Published:** 1898-01-22

**Authors:** 


					j THE HOSPITAL. ? The standard of highest purity.''?Lancet. Jan. 22nd, 189s;
CADBURY'S ^ 1U im CAR?URY'S
COCOA 1\ fFll COCOA
ABSOLUTELY PURE. DRUGS OR ALKALIES USED,
RWICH HOSPI TAL
? ASrniv l?riRMaRY
_ 591-Yo1- mn. PlSSitf satubday,
Jan. 22nd, 1898.
[BubscriptionTonns,] pRICE TWOPENCE.

				

